# Editors’ Book Table

**Published:** 1874-05

**Authors:** 


### Editors' Book Table.

NOTE.—All works reviewed in the columns of the CHICAGO MEDICAL JOURNAL may be found in the extensive stock of W. B. KEEN, COOKE & Co., whose catalogue of Medical Books will be sent to any address upon request.

*Physicians' Pocket Case-Record, and Prescription Blank Book.* Cincinnati Case-Record Company. 1874.

Is a form for the writing and registration of prescriptions, and the recording of clinical memoranda. The Office Case-Record, etc., being a quarto edition of the same, both arranged somewhat after the manner of a check book, the leaf being of sufficient length to contain the prescription and a duplicate or abstract thereof upon the stub, after the prescription shall have been torn off; on the reverse side of the stub is a blank for recording the patient's name, address, diagnosis, age, build, pulse, tongue, urine, stools, heat, etc. All this is very useful, but involves an amount of clerical labor which few physicians are willing to perform. It will doubtless find its admirers who will use it, for awhile, and when the novelty wears off will abandon it.

One feature of the system we must condemn; it is copyrighted. We contend that every member of the medical profession is bound in honor to contribute everything in his power to the advancement of his profession, and has no right to trade upon the necessities of his professional brethren for his own exclusive benefit. We have no intention to impugn the motives of the author of this work, who has doubtless acted in good faith according to his view of the matter. Fancy Laennec patenting his stethoscope, or Hodge his forceps! If we have anything to offer to our profession which may accrue to its advantage, let us give it freely, and trust to our reward in our participation in the general advancement. H.

*Clinical Researches in Electro-Surgery.* By A. D. ROCKWELL, A.M., M.D., and GEO. M. BEARD, A.M., M.D. New York: William Wood & Company. 1873.

A really valuable contribution to the resources of clinical surgery; being an apparently honest report of experiments for the removal, by both the electrolytic and galvano-caustic methods, of erectile and vascular, cystic and fibrous, epithelial and scirrhus growths, showing the actual and relative advantages of the two methods. The results of these experiments have been highly satisfactory to the authors, and seem to have demonstrated to them the exemption of these methods from some of the objections usually urged against them, among which may be mentioned pain, which seems by no means a necessary accompaniment of the application of these methods of treatment.

The second chapter comprises the results of experiments made for the relief of various forms of skin disease, in which both local and central galvanization has been applied. The results in this department of clinical research have been quite as satisfactory as in the last one, and, moreover, seem to rest upon more certain physiological data. Eczema, prurigo, acne, pityriasis, psoriasis, and even elephantiasis, have been made the subjects of experiments, and in all cases the results have justified the belief that, to say the least, a very valuable addition has been made to the armamentum chirurgicum in the galvanic battery, and a very important contributon to our knowledge of its application by thi [illegible] t treatise of Drs. Rockwell and Beard, which will amply compensate any one who may desire information upon these subjects, for the money and time spent in its purchase and perusal. H.

*Lectures on the Clinical Uses of Electricity.* Delivered in the University College Hospital. By J. RUSSELL REYNOLDS, M.D., F.R.S., Fellow of the Royal College of Physicians, etc., etc. Second edition. Philadelphia: Lindsay & Blakiston. 1874.

The study of, and the general interest awakened in, the therapeutic relations of the various forms of electricity, seem destined to initiate a new era in the history of practical medicine.

Anything emanating from the pen of J. Russell Reynolds, to whom the students of medicine are already so much indebted, is entitled to thoughtful study, and the little volume before us is not an exception to this general commendation. The lectures, five in number, with an appendix, have already been published in the London *Lancet.*

The author is one of the very few who can speak with authority, and yet not dogmatically. This is well illustrated in the first division, in which the diagnostic and the therapeutical capacities of electricity are analyzed and limited; and in the second, in which the various forms of this force are defined and their applicability explained. The tyro in electro-therapeutics will find this little book one of the clearest, most explicit and intelligible guides to a correct understanding of the real value of this most important agent; and the adept will derive that pleasure from its perusal which an expert always finds in the work of a master hand. H.

*The Student's Guide to Surgical Anatomy.* Being a description of the most important surgical regions of the human body, and intended as an introduction to operative surgery. By EDWARD BELLAMY, F.R.C.S., Associate of King's College, London, etc. With illustrations. Philadelphia: Henry C. Lea. 1874.

The author, in his preface, comments very justly upon the paucity, in English medical literature, of works similar to his own, and assumes with equal justice that his little book will serve to supply a need which all students must have appreciated. He has done his work well, for it is rare to find so much practical knowledge condensed into the small compass of six chapters. The plan of the work is simple, the style concise, and the matter practically important. The illustrations, about fifty in number, are for the most part taken from reliable sources, about one-third being original. Taken altogether, this is one of the most useful little books accessible to English-speaking students, and there are few whose studies of surgical anatomy will not be materially facilitated by it. H.

*The Physician's Dose and Symptom Book;* containing the doses and uses of all the principal articles of the Materia Medica, and officinal preparations, etc., etc. By JOSEPH H. WYTHES, A.M., M.D., etc. Eleventh edition, revised. Philadelphia: Lindsay & Blakiston. 1874.

What we have said on a preceding page of another little book, whose author's name bears a striking resemblance to that of the compiler of the little work before us, will apply with equal force to this.

We have no fault to find with the good intentions of the author, which he says, in his preface, were "to save trouble," and that his intentions have been abundantly appreciated by large numbers of physicians willing to "save trouble," is demonstrated by the fact that ten editions of the work have been already exhausted. We cannot avoid expressing the belief that the general use of such books tends to encourage superficiality, already the greatest defect in medical education, and to lower the standard of professional attainment, already far too low. The excuse for the publication of these "ready reckoners," and short cuts to knowledge, time-honored since the days of Neill & Smith's Compendium and Mendenhall's Vade Mecum, both long since, happily, consigned to oblivion, *i. e.*, to save trouble of reference by the busy practitioner, is a fallacy, as the busy practitioner carries, or should carry, the contents of such a book in his own memory, compiled from the records of his daily experience. In fact, they are not found in such hands, but in those of medical students trying to supplement the idleness and negligence which marked their "first course" by "cramming" for examination; or in after life, "posting up" in an emergency.

The matter of the book is scarcely up to the times. *Ex. gr.:* to an inquirer anxious to "post up" on bromide of potassium, the information, "Use as iodide of potassium, but slower in effect," will be of questionable value. The dose of tincture of aconite (leaves or root?) prescribed, *i. e.*, "10 to 40 drops, gradually increased," would not only speedily cure a case of "neuralgia or rheumatism," but effectually prevent its return, in the same patient and probably in the physician. The practitioner who prescribes $\frac{1}{16}$ to $\frac{1}{12}$ gr. of strychnia, as here directed, should study well the phenomena of tetanus, to guard against surprises. Nor do we think that the alterative effects of prot-iodid of mercury will be best demonstrated by doses of ij. grs.

*Lectures on Bright's Disease, with especial reference to Pathology, Diagnosis and Treatment.* By GEO. JOHNSON, M.D., F.R.S., Fellow of the Royal College of Physicians; Honorary Fellow of King's College, London; Professor of Medicine in King's College, and Physician to King's College Hospital. New York: G. P. Putnam's Sons. 1874.

Twenty-eight years have elapsed since the publication of his papers in the Medico-Chirurgical Transactions identified the name of George Johnson with the study of renal pathology, and his Gulstonian Lectures in 1852, and the publication of his treatise on Diseases of the Kidney, in the same year, marked him as one of the greater teachers in this department of medicine, and enrolled his name with those of Bright, Garrod, Golding, Bird, Bence, Jones, Todd, and Rayer. This last contribution to the literature of renal disease in no way detracts from the author's high reputation. The work before us consists of seven lectures, arranged in as many chapters, of which the first comprises the minute anatomy and physiology of the kidney and the general pathology of Bright's disease, the latter of which the author formulates into five propositions, viz.:

I. Bright's disease is not merely a local malady, but a disease of constitutional origin; and the proximate cause of the renal disease is, in all probability, a morbid condition of the blood. * * *

II. The morbid blood, which is assumed to be the proximate cause of Bright's disease in all its forms, exerts its influence primarily upon the gland-cells which line the convoluted tubes. * * *

III. The structural changes which occur in the basement-membrane and in the malpighian capsules are direct results of intra-tubular cell-changes.

IV. During the progress of chronic Bright's disease, the blood-vessels in the kidney and in many tissues and organs undergo very interesting changes, but these occur later and less constantly than those which affect the secreting tissues of the gland.

V. The pathological products of the structural changes within the tubes, being carried out by the liquid secretion, escape with the urine and appear in the form of cylindrical casts of the uriniferous tubes; and a microscopical examination of these tube-casts affords most interesting and valuable information as to the nature and the stage of the renal disease.

The second chapter treats of acute Bright's disease under its various synonyms, acute desquamative nephritis, acute albuminuria, and acute renal dropsy; the form of this disease frequently occurring as one of the sequelæ of scarlet fever being assumed as the type; acute Bright's disease, however, may occur without desquamation, without albuminuria, and permits, generally, a favorable prognosis.

The third, fourth and fifth chapters constitute a complete treatise upon chronic Bright's disease under its different forms, marked by the small, contracted and granular, and the large white or fatty, and the lardaceous or waxy kidney, together with the organic complications arising in its course.

In this, the chronic, form of the disease, the author gives us little to hope for in the way of favorable prognosis, regarding every variety of it as essentially fatal.

The sixth lecture, upon the subject of albuminuria unassociated with Bright's disease, possesses much interest, inasmuch as the difficulties perceived by some in distinguishing these morbid phenomena may therein be entirely cleared away. The section relating to puerperal albuminuria will be found especially clear and concise.

The treatment of both forms of the disease is detailed in the seventh and last chapter, and is an admirable illustration of physiological therapeutics, quite refreshing in this age of empiricism.

BOOKS RECEIVED.

*The Marine Hospital Service of the United States from* 1798 *to* 1871; being the Annual Report of the Supervising Surgeon for the fiscal year 1873. By JNO. M. WOODWORTH, M.D.

*Treatment of Nervous and Rheumatic Affections by Static Electricity.* By Dr. A. ARTHIUS. Translated from the French by I. H. Etheridge, M.D., Professor of General Therapeutics, Rush Medical College, Chicago. W. B. Keen, Cooke & Co. 1874.

*Remarks on the Management of Intermaxillary Bone in Double Hair Lip.* By W. R. WHITEHEAD, M.D., of Denver, Colorado. Denver, 1873.

PAMPHLETS RECEIVED.

*Report of the Autopsy of the Siamese Twins*, together with other interesting information concerning their life, reprinted from the *Philadelphia Medical Times.* Philadelphia: J. B. Lippincott & Co. 1874.

*Epidemic Diseases as dependent upon Meteorological Influences.* By C. SPINZIG, M.D. St. Louis, Mo., 1874.

*Report of the Vaccine Department of the New York Dispensary for the year* 1873. By Dr. FRANK P. FOSTER, Director of the Vaccine Department. New York, 1874.

*Twenty-fifth Annual Report of the Indiana Hospital for the Insane*, for the year ending October 31, 1873, to the Governor. Indianapolis, 1874.

*Contributions to the Study of Yellow Fever.* By J. M. TONER, M.D., and JNO. M. WOODWORTH, M.D. Washington, 1874.

*Constitution and By-Laws* of the Association of the Alumni of the Albany Medical College. Albany, 1874.

*Constitution and By-Laws* of the Medical Society of Central Missouri. Jefferson City, Mo., 1874.

*Cerebro-Spinal Meningitis in Massachusetts in* 1873, with some Inquiry into the Circumstances Attending its Origin and Supposed Cause. By J. BAXTER UPHAM, M.D. Boston, 1874.

*Writers' Cramp or Scriveners' Palsy.* By REUBEN A. VANCE, M.D., New York.

*Skin Grafting.* By J. R. LEWIS, M.D., Surgeon to the Pennsylvania Hospital, and to Will's Ophthalmic Hospital.

JOURNALS RECEIVED.

L'Anatomie et de la Physiologie, Journal de. CHARLES ROBIN. Paris.—No. 1, Janvier et Fevrier; No. 2, Mars et Avril, 1874.
Archives of Ophthalmology and Otology. Knapp & Roos. New York and Heidelberg. Vol. III, No. 2.
American Practitioner, Louisville, Ky.—April, 1874.
American Journal of Syphilography and Dermatology—April, 1874.
Atlanta Medical and Surgical Journal—April.
The Boston Medical and Surgical Journal—April, 1874.
" British Journal of Dental Science—Feb. and March, 1874.
" Buffalo Medical and Surgical Journal—Feb., March and April.
" Boston Journal of Chemistry—April.
" Canada Medical and Surgical Journal—March and April.
" Clinic, Cincinnati—March 21, 28, April 4, 11.
" Cincinnati Lancet and Observer—April.
" Dental Cosmos—April, 1874.
" Eclectic Medical Journal, Cincinnati—April, 1874.
" Indiana Journal of Medicine—March and April, 1874.
" Kansas City Medical Journal—March and April, 1874.
" London Lancet—March, 1874.
" Medical Examiner, Chicago—March 15, April 1.
" Medical and Surgical Reporter, Philadelphia—March 21, 28, April 4.
" Medical Times, Philadelphia—March 21, 28, April 4.
" Medical Times and Gazette, London—March 7, 1874.
" Missouri Clinical Record, Vol. 1, No. 1, St. Louis, Mo.—April, 1874.
" Medical Record, New York—April, 1874.
" Medical Press and Circular, London—March 18, 1874.
" Medical News and Library—April, 1874.
" Medical Herald, Leavenworth, Kansas—April, 1874.
" Medical Union—New York, April, 1874.
" New York Medical Journal—April, 1874.
" North-Western Medical and Surgical Journal—April, 1874.
" Nashville Journal of Medicine and Surgery, March, 1874.
" Ohio Medical and Surgical Reporter—January and March.
" Pacific Medical and Surgical Journal, San Francisco—March and April.
" Physician and Pharmacist, New York—March, 1874.
" Progres Medicale, Nos. 6, 7, 8, 9, 10, Paris, 1874.
" Pharmacist, Chicago—April, 1874.
" Practitioner, London—March, 1874.
" Richmond and Louisville Medical Journal—March and April, 1874.
" St. Louis Medical and Surgical Journal—April, 1874.
" Southern Medical Record, Atlanta—March, 1874.

